# Scoring systems for the Clock Drawing Test: A historical review

**DOI:** 10.1590/1980-57642016dn11-010003

**Published:** 2017

**Authors:** Bárbara Spenciere, Heloisa Alves, Helenice Charchat-Fichman

**Affiliations:** 1 BsC, Department of Psychology, Pontifical Catholic University of Rio de Janeiro RJ – Brazil.; 2 PhD, Department of Psychology, Pontifical Catholic University of Rio de Janeiro RJ – Brazil.

**Keywords:** Clock Drawing Test, scoring, neuropsychology, screening test

## Abstract

The Clock Drawing Test (CDT) is a simple neuropsychological screening instrument that is well accepted by patients and has solid psychometric properties. Several different CDT scoring methods have been developed, but no consensus has been reached regarding which scoring method is the most accurate. This article reviews the literature on these scoring systems and the changes they have undergone over the years. Historically, different types of scoring systems emerged. Initially, the focus was on screening for dementia, and the methods were both quantitative and semi-quantitative. Later, the need for an early diagnosis called for a scoring system that can detect subtle errors, especially those related to executive function. Therefore, qualitative analyses began to be used for both differential and early diagnoses of dementia. A widely used qualitative method was proposed by Rouleau et al. (1992). Tracing the historical path of these scoring methods is important for developing additional scoring systems and furthering dementia prevention research.

## Abstract (pt)

O Teste do Desenho do Relógio (TDR) é um instrumento de rastreio neuropsicológico simples bem aceito pelos pacientes e com sólidas propriedades psicométricas. Em meio aos métodos de pontuação, não existe consenso com relação aos que tenham maior acurácia. Esse artigo tem como objetivo realizar uma revisão histórica sobre os sistemas de pontuação mais utilizados e as mudanças ocorridas com os mesmos ao longo dos anos. No decorrer do caminho histórico, diferentes tipos de sistemas de pontuação surgiram. Inicialmente, o foco era no rastreio de demência e os métodos utilizados eram quantitativos e semi-quantitativos. Contudo, em um segundo momento, a necessidade de diagnóstico precoce demandou o uso de um sistema de pontuação que pudesse especificar erros sutis especialmente aqueles relacionados a funções executivas. Assim, análise qualitativa começou a ser mais usada em ambos no diagnóstico diferencial e precoce de demência. Um método qualitativo amplamente utilizado é o de Rouleau et al. (1992). O caminho histórico é importante para o desenvolvimento dos sistemas de pontuação e também para as pesquisas de prevenção de demência.

## INTRODUCTION

The Clock Drawing Test (CDT) is recognized worldwide as a neuropsychological screening test that has solid psychometric properties, including test-retest reliability^1,2^ and inter-rater reliability.^3-6^ The CDT correlates well with other instruments, such as the Mini-Mental State Examination (MMSE), Cambridge Cognitive Examination (CAMCOG), and Rey Complex Figure Test, among others.^1,7-12^ It is usually used to screen for cognitive decline in older adults and discriminate healthy individuals from those with dementia, especially Alzheimer's disease (AD). The CDT is well accepted by patients and widely employed as a follow-up instrument because it can be easily and quickly applied and scored.^13^

While drawing the clock, different cortical systems work simultaneously, including the frontal, parietal, and temporal lobes.^12,14,15^ Thus, different cognitive abilities can be measured, such as selective and sustained attention, auditory comprehension, verbal working memory, numerical knowledge, visual memory and reconstruction, visuospatial skills, on-demand motor execution (praxis), and executive function.^1^

Some features influence the CDT, such as age and education. A number of authors also consider language an influential factor.^16^ Older adults usually exhibit more impaired performance than young adults,^12,17-19^ and greater years of formal education are associated with better performance on the test.^18-21^

Clock Drawing Test performance was originally used as an indicator of constructional apraxia. During World War II, it was employed in studies of soldiers who were victims of head trauma and had possible focal lesions in the occipital and parietal lobes. Goodglass and Kaplan^22^ conducted the first systematic study of the CDT as part of the Boston Aphasia Battery.^13^

Although the CDT had been mainly used to assess visuoconstructional disorders, clinical staging scales for the study of AD began to be developed in the 1980s to provide a more specific classification of intellectual performance in the geriatric population.^23^ In August 1986, Shulman and collaborators published the first study employing the CDT as a screening tool for older adult patients with cognitive disorders.^14^ Since then, studies have been performed to characterize its contributions both as a screening instrument in cognitive impairment and for the diagnosis and follow-up of Huntington's disease, schizophrenia, unilateral neglect, delirium, multiple sclerosis, and other pathologies.^13^

Different studies have used a wide range of administration and scoring criteria, resulting in heterogeneous findings and a lack of consensus with regard to which criteria produce the best results. In this context, the need for putting together a logical timeline that better explained the development of CDT scoring systems emerged. Taking into account the large number of studies since 1986, a selection criterion based on relevance was indispensable. The articles were selected based on their importance to the development of CDT scoring systems throughout history. Therefore, the articles that met the criteria of representing a significant milestone and/or change in the development of CDT scoring systems were included. Finally, the aim of this article was to conduct a historical literature review of CDT scoring systems, describing the development and changes that have taken place throughout the years.

## SCORING SYSTEMS CLASSIFICATIONS

Shulman^13^ highlighted that no matter which method is employed, the specificity and sensitivity of the CDT depend more on the interpretation of the test than the way the test is administered. The presentation of different scoring procedures is intended to provide clinicians with as much information as possible about these procedures. However, because reviewing all of these in detail would be beyond the scope of the present study, we focus instead on the tendencies and changes that the scoring systems have undergone over the years.

Different methods of scoring the CDT emerged, and the classifications of scoring systems often diverged. For the purposes of the present study, our classifications were based on Ehreke et al.^24^ and Patocskai et al..^25^ These authors considered quantitative analyses as those represented by numerical scales. Qualitative analyses classify the drawing of the clock based on descriptions of typical errors by considering the whole clock in their analysis and using a subjective approach.^24,25^ Semi-quantitative systems also utilize a subjective approach, in which the whole clock is analyzed, but a numerical scale is used to characterize a quantitative domain. Two examples are the methods proposed by Sunderland et al.^10^ and Shulman et al..^26,24^ Table 1 illustrates the different classifications of CDT scoring systems and the chronological changes they have undergone over the years.

**Table 1:** Different quantitative, semi-quantitative, and qualitative CDT scoring systems throughout the years.

Method	Type	Specificities
Shulman et al.^29,26^*	Semi-quantitative	Hierarchical scale
Sunderland et al.^10^	Semi-quantitative	Hierarchical scale
Wolf-Klein et al.^30^	Semi-quantitative	Visual clock patterns
Mendez et al.^1^ (Clock Drawing Interpretation Scale)	Quantitative	Aspects evaluated separately
Rouleau et al.^11^	Quantitative	Aspects divided into categories
	Qualitative	Evaluation of specific error aspects
Tuokko et al.^31^	Quantitative	Distribution of error types into categories
Watson et al.^32^	Semi-quantitative	Division of clock into quadrants
Death et al.^33^	Semi-quantitative	Hierarchical scale
Manos and Wu^9^	Quantitative	Division of clock into eighths
Freedman et al.^12^	Quantitative	Ordinal scale with categories
Todd et al.^34^	Quantitative	Division of error types into categories
Cahn et al.^35^	Quantitative	Evaluation of aspects in categories
	Qualitative	Evaluation of specific error categories
Libon et al.^36^	Semi-quantitative	Hierarchical scale
	Qualitative	Evaluation of specific error categories
Lam et al.^37^	Semi-quantitative	Hierarchical scale
Royall et al.^38^	Quantitative	Evaluation of aspects separately
Borson et al.^39^	Semi-quantitative	Hierarchical scale
Cacho et al.^40^	Quantitative	Division of aspects into categories
Jitapunkul et al.^41^	Quantitative	Division of clock into quadrants
Lin et al.^42^	Quantitative	Evaluation of aspects separately
	Short version	Simplified scale with three items
Heinik et al.^8^	Quantitative	Division of aspects into categories
Freund et al.^43^	Quantitative	Division of aspects into categories
Babins et al.^44^ (18-point)	Quantitative	Division of error types into categories
Lessig et al.^45^	Quantitative	Division of error types into categories
Leyhe et al.^46^	Semi-quantitative	Hierarchical scale
	Qualitative	Evaluation of specific error categories
Parsey and Schmitter-Edgecombe^47^	Quantitative	Division of aspects into categories
	Qualitative	Evaluation of specific error categories
Kim et al.^48^	Quantitative	Indication of range of dementia
		Observation of qualitative features
Juok and Tuokko^49^	Quantitative	Division of typical error categories
Nyborn et al.^50^	Qualitative	Long scale of features and types of errors
Wang et al.^51^	Quantitative	Division of aspects into categories
Ricci et al.^52^	Quantitative	Division of aspects into categories

* This method was reviewed in 1993 and therefore not placed chronologically in the table.

Initially, the scoring systems used a semi-quantitative approach (Table 1). The CDT was then used as a medical screening tool to diagnose cognitive deficits in dementia and delirium. No interest was expressed in specific aspects of errors made during the CDT because it was simply used to differentiate healthy older adults from those with dementia.

The CDT meets the criteria for a cognitive screening instrument. It is quick to apply, well accepted by patients, easy to score, and relatively independent of language, education, and culture. It also has good inter-rater and test-retest reliability, high levels of sensitivity and specificity, concurrent validity, and predictive validity. Because of these attributes, it has seen widespread clinical use.^13^

Shulman^13^ reported mean levels of sensitivity and specificity of 85% among all scales analyzed from 1983 to 1998. Even so, because of difficulties in replicating the results, conceptual disagreement is still seen in the literature. In Brazil, Lourenço et al.^20^ and Aprahamian et al.^27^ compared different scoring methods and found they were equivalent. Although previous studies demonstrated this, opinions regarding which method should be adopted for the test's interpretation are far from reaching a consensus. Authors highlight the methods proposed by Shulman et al.,^26^ Sunderland et al.,^10^ and Mendez et al.^1^ as the most accurate ones.^13,28^

## HISTORICAL TRAJECTORY MILESTONES

Shulman et al.^29^ proposed one of the first scoring systems which remains one of the most widely used scales in the literature to this day.^7^ It consists of a hierarchical scale (i.e., a scale with severity ratings), in which the clock is analyzed as a whole. The system was reviewed in 1993.^13^

Three years later, two other authors published new methods: Sunderland et al.^10^ and Wolf-Klein et al..^30^ Sunderland's method is still widely employed. It is more detailed and has categories of typical errors.^16^ Wolf-Klein et al.^30^ used visual clock patterns instead of a hierarchical scale.

An increasing use of screening tests was evident, especially with older adults. However, during a second period in history, the use of quantitative scoring systems became more frequent, and more objective scoring methods were sought. Using a quantitative approach, the CDT could be scored faster and easier so was more readily applied by busy clinicians. At this point in time, the quantitative scoring system was important for the early identification and monitoring of dementia because of the growing number of older adults and high prevalence of cognitive impairment among them.

The scoring system proposed by Mendez et al.^1^ was a quantitative scale, referred to as the Clock Drawing Interpretation Scale (CDIS).^16^ Similar to Sunderland's system, Mendez's system was also based on the frequency of errors committed in the CDT.^53^ However, it did not take the whole clock into account. Instead, the evaluation was performed separately, focusing on one aspect of the clock at a time.^24^

In the same year, Tuokko et al.^31^ developed a quantitative scoring system that demanded more from the participant compared with other systems.^14^ It consisted of three stages: clock drawing, setting, and reading.^16,51^ This way of evaluating the drawing allowed the analysis of different cognitive abilities and comparisons of performance in each stage. This was necessary because constructional skills tend to decline in normal aging while abstract conceptualization (i.e., executive function) is preserved. The diagnosis of dementia depends in part on abstract thinking and reasoning, which are also important for clock reading and setting. Therefore, the method scored performance efficiency in general and also specific types of errors.

Quantitative scoring systems for the CDT are especially useful in moderate and severe dementia.^52^ Powlishta et al.^54^ suggested that mild dementia, particularly AD, can also be differentiated from normal aging based on the CDT, but its sensitivity for detecting very mild dementia is poor. This aspect needs to be highlighted because it can cause sub- or even misdiagnoses.^54^ The CDT is also not very useful for identifying individuals with mild cognitive impairment (MCI) because it does not allow descriptions of the participants' error profiles.^55^ At this point, the medical approach was no longer sufficient for detecting the transition from normal to pathological aging. It was necessary to refine and improve the method using a neuropsychological approach that analyzes information processing and its qualitative aspects. Understanding the ways in which older adults draw the clock solely by considering the final score became insufficient for differentiating groups. It became important to evaluate the executive functions involved in the task and the execution of the drawing by analyzing errors.

Qualitative approaches became more useful by analyzing error types and thus helping describe different dementia profiles.^11,56^ As such, qualitative scoring systems are helpful for differential diagnoses.^56^

The first study that used a qualitative scoring method was by Rouleau et al..^11^ To better differentiate cognitive deficits into two progressive types of dementia (i.e., Huntington's disease and AD), the authors used both quantitative and qualitative methods. The method proposed by Rouleau et al. was similar to the one used by Mendez et al..^1^ It also analyzes specific aspects of the clock separately. The quantitative part has three independent subscales: clock face, numbers, and hands. The qualitative error analysis proposed by Rouleau had six categories: clock size, graphic difficulties, stimulus-bond response, conceptual deficit, spatial and/or planning deficit, and perseveration.^11,53^

Executive function domains, such as abstract conceptualization, planning, and cognitive flexibility, began to be analyzed. Visuoconstructional skills can decline in normal aging, but abstract conceptualization may remain intact.^31^ Although Tuokko et al.^31^ also highlighted abstract conceptualization, their scoring method did not thoroughly describe the patients' profiles as Rouleau's^11^ qualitative scoring system did.

Parallel to the evolution of CDT scoring methods at this point in history, a controlled trial of the cholinesterase inhibitor tacrine in AD produced results that confirmed its safety and efficacy for AD treatment. It was the first medication approved by the United States Food and Drug Administration for the treatment of cognitive deficits in AD.^57^ However, although the medication had beneficial effects on cognition, it did not slow progression of the disease. The need for an earlier diagnosis of dementia was clear, and targeting early stages of dementia became fundamental. Starting treatment as soon as possible increased the chances of slowing the progression of the disease.^58^

For such early diagnoses, it was necessary to analyze specific error patterns in clock drawing, especially with regard to executive function. The qualitative scoring systems of the CDT are better able to identify early cognitive decline,^55^ and such qualitative methods became the most widely employed.

Until 1998, although the types of scoring systems were changing, the main focus of the CDT was still visuoconstructive ability (Table 2). Although the CDT was considered a sensitive measure of "abstract thinking" and "complex behavior," no distinction was made between constructional errors and executive function errors.^38^ However, in 1998, Royall et al.^38^ created a new way of scoring the CDT that significantly impacted the historical evolution of the CDT, called CLOX. It was a quantitative method designed to specifically evaluate executive function.^16,53^

**Table 2:** Historical changes in CDT scoring systems.

Scoring system	Type	Focus
Shulman et al.^29,26^	Semi-quantitative	Visuospatial disorganization
Sunderland et al.^10^	Semi-quantitative	Visuospatial ability
Mendez et al.^1^	Quantitative	Constructional skills
Tuokko et al.^31^	Quantitative	Abstract conceptualization (reading and setting)
		Constructional skills (drawing)
Rouleau et al.^11^	Quantitative	Visuoconstructive impairment
	Qualitative	Executive function
Lam et al.^37^	Semi-quantitative	Analysis of constructional skills
Royall et al.^38^	Quantitative	Analysis of executive function

Alzheimer's disease affects temporal cortical regions before it affects the frontal cortex. Isolated executive function errors do not indicate early AD. Many non-AD diseases, such as other "reversible" dementias, might be more expected to produce executive function impairment.^38^ A scoring system that differentiates executive function and constructional skills is very important for the diagnosis of the different subtypes of MCI and also early stages of dementia.

Qualitative types of scoring continued to emerge. In 2000, Shulman^13^ suggested that using a simple scoring system that emphasizes the qualitative aspects of clock drawing, together with a quantitative system, could maximize the utility of the test.

In 2011, Parsey and Schmitter-Edgecombe^47^ demonstrated the accuracy of the CDT in distinguishing MCI, AD, and normal aging. Their qualitative scoring system was based on Rouleau et al..^11^ Combined with evaluations of error types, it became even more sensitive for the detection of MCI. It also highlighted the importance of qualitative scoring systems in the early identification of cognitive decline.^55^

Today, there is an increasing tendency to use qualitative methods together with either quantitative or semi-quantitative methods. The emergence of more qualitative scoring systems^47,56^ is attributable to the fact that solely quantitative methods are unable to describe subjects' error profiles or specific cognitive changes the way that qualitative studies can.^55^

Another recent trend is that some scoring methods are beginning to be computerized.^48,59^ Such computerized methods analyze the entire construction process and not only the final drawing. Computerized systems enable the assessment of qualitative features, but the resulting score only indicates the presence or absence of dementia. Because the main goal is to screen for dementia, there is no qualitative score, and it does not thoroughly specify the neuropsychological characteristics of the drawing.^48^

## SPECIFICITIES AND APPLICABILITY OF DIFFERENT SCORING SYSTEMS

As presented in Table 3, different types of CDT scoring systems have advantages and disadvantages. The best method to be applied depends on the specific situation.

**Table 3:** Advantages and disadvantages of different types of scoring systems.

Type of method	Advantages	Disadvantages
Semi-quantitative	Fast scoring Screens severe and moderate dementia	Subjective Does not measure specific errors Does not differentiate diagnostic groups
Quantitative	Objective Fast scoring Screens severe and moderate dementia	Does not measure specific errors Does not differentiate diagnostic groups
Qualitative	Measurement of specific errors Differential diagnosis Distinguishes different ranges and types of dementia	Highly time consuming Subjective

Several studies,^28,54,60-63^ including Brazilian ones,^20,27^ have compared different CDT scoring methods. These comparisons usually evaluated reliability, correlations, and the importance of the instrument compared with other tests in the diagnosis of dementia in older adults. These studies concluded that the CDT can be scored reliably using a variety of scales and can accurately discriminate healthy individuals from older adults with dementia.

Because of the need for more specific and early diagnoses of pathologies and impairments, research on the preclinical stages of dementia, especially in AD, is increasingly focusing on the early detection of cognitive decline. It becomes crucial to characterize the cognitive profile that better predicts the progression from preclinical to clinical stages of the different subtypes of MCI and AD. Charchat-Fichman et al.^64^ highlight the diagnostic heterogeneity among transitional stages to dementia and the importance of understanding this heterogeneity for an earlier and more precise diagnosis.

Studies suggest that minimal cognitive alterations can be detected even before the criteria for MCI are met, thus enabling better predictions of possible progression to AD. However, the neuropsychological tests and scoring systems employed currently are still unable to detect such early stages.^65^ Amodeo et al.^66^ stated that the CDT is useful for the longitudinal assessment of cognitive impairment. These authors also contended that, together with other tests, the CDT can predict conversion to dementia.

Other authors have further suggested that the analysis of a multidimensional test, such as the CDT, based only on a single numerical score tends to impair both the sensitivity and specificity of the instrument.^67^ Therefore, a single-score quantitative method may not be able to detect subtle differences between healthy individuals and subjects with mild forms of cognitive impairment.^24^ This specific approach also compromises the ability of the test to discriminate between different error types, which is imperative for differentiating AD from other cognitive disorders. Thus, exclusively using quantitative scoring methods does not yield the desired results, increasing the need for more studies on qualitative methods.^5^

Some studies have demonstrated the advantages of qualitative scoring methods for the CDT. Parsey and Schmitter-Edgecombe^47^ and Fabricio et al.^55^ differentiated diagnostic groups using a qualitative method that could not have been achieved with quantitative analysis. Mendez et al.^68^ also illustrated that the analysis of specific errors, rather than global performance in clock drawing, is helpful for differentiating early-onset AD, behavioral variant frontotemporal dementia, and other conditions. Qualitative features were also more helpful than quantitative features for localizing lesion sites and differentiating subcortical and cortical cases of stroke.^69^

## CONCLUSION

The historical literature search performed in the present study revealed a great number of CDT scoring systems. Throughout the years, these different systems have changed and improved in several ways,^53^ but direct comparisons between methods are often difficult because no consensus has been reached concerning the different methods.

It is generally agreed that the CDT is useful for detecting moderate and severe stages of dementia. Its ability to characterize MCI or even mild dementia is still controversial. There are divergent opinions regarding the different scoring systems because some are too complex and time consuming while others are too simplistic to have sufficient sensitivity and specificity.^52^

In Brazil, different types of CDT quantitative methods are used^19^ and studies conducted considering the role of formal education in the test.^70,71^ However, few studies have employed qualitative scoring methods.^55^ Given the dearth of studies and the importance of the CDT for aging research, more studies on qualitative analyses of the CDT should be performed.

Another important aspect that should be more frequently addressed is the role of executive function. In Brazil, the CDT is used in the assessment of executive functions.^71^ Paula et al.^19^ highlighted in their study the importance of executive functions in CDT performance. However, as shown in the present historical review, very few studies have focused on this particular topic. Scoring methods usually consider the types of errors and specific features of the drawing, but none of them evaluate the planning strategies used by subjects during execution of the drawing.

A wide range of studies have presented different CDT scoring systems, since 1986, when it was first used as a screening instrument. This large number precluded discussion of all methods in this study, where the limitation of the study was failure to describe all of them. Thus, the articles were selected based on their historical importance. On the other hand, the study suggests possible future studies on CDT scoring criteria with a focus on executive functions and also as a system for the specific evaluation of planning strategies.

In summary, historically rooted discussions of the CDT and a better path to improving its scoring methods are warranted. Different clinical needs and circumstances throughout history have molded this path and led to the present tendencies in scoring methods. Despite the advantages and disadvantages of each scoring method, the current tendency of employing both quantitative/semi-quantitative and qualitative approaches provides a better understanding of patients and more precise diagnoses. Qualitative aspects complement quantitative ones, making the CDT a complex tool for investigating cognition during the aging process. Although qualitative analyses require specific training, their inclusion into neuropsychological assessments is appropriate and much needed.
